# Great Britain

**Published:** 1896-04

**Authors:** 


*Great Britain.* A bill has been introduced in the House of Commons to amend the Diseases of Animals Act of 1894, with the object of abolishing the discretion now enjoyed by the Minister of Agriculture to admit foreign cattle and to make the present restrictions permanent.

The *Veterinary Journal*, fully alive to the needs of the hour in helping the movement to provide a pure and wholesome supply of milk to the people of Great Britain, calls for increased cleanliness in the animals used for dairy purposes; all milking to be carried on in the open air, on a concrete or a cement floor; the hands and clothing of the milkers to be clean and free from dirt of any kind; the places of the milk-vendors to be cleaned, with proper storage facilities, and counters for its dispensing tiled. All of which will come to pass soon at the latest, and why not before in the handling of this raw product, so fraught at times with dangers direct and indirect, seems in these days incomprehensible.

The Agricultural Produce Bill passed second reading in Parliament on the 18th of March, providing for the marking of foreign and colonial meat and for the registration of persons who deal in the same. One member advocated the marking of American cheese.

